# Circulating Angiogenic Factors in Patients with Thromboangiitis Obliterans

**DOI:** 10.1371/journal.pone.0034717

**Published:** 2012-04-10

**Authors:** Bernd Hewing, Verena Stangl, Karl Stangl, Kathrin Enke-Melzer, Gert Baumann, Antje Ludwig

**Affiliations:** 1 Medizinische Klinik mit Schwerpunkt Kardiologie und Angiologie, Campus Mitte, Charité - Universitätsmedizin Berlin, Berlin, Germany; 2 Klinik für Gefäßmedizin, Helios Klinikum Emil von Behring, Berlin, Germany; University of Bristol, United Kingdom

## Abstract

**Background:**

Thromboangiitis obliterans (TAO, also known as Buerger's disease) is a non-atherosclerotic inflammatory vascular disease that primarily affects arteries in the extremities of young adult smokers. Since the etiology of TAO is still unknown, therapeutic options are limited. Recent attempts in therapeutic angiogenesis have been promising. Therefore, the aim of our study was to evaluate angiogenic processes and factors including circulating progenitor cells in TAO.

**Methodology/Principal Findings:**

TAO patients with critical limb ischemia and age- and gender-matched nonsmokers and smokers without cardiovascular disease (n = 12 in each group) were enrolled in the study. Flow cytometric analysis of peripheral blood showed significantly decreased levels of circulating CD45^dim^CD34^+^ progenitor cells in TAO patients and in smokers compared to nonsmokers. In contrast to both control groups, the proportion of CD45^dim^CD34^+^ progenitor cells co-expressing VEGF receptor-2 (VEGFR2) was significantly elevated in TAO patients. Enzyme-linked immunosorbent assay (ELISA) of common angiogenic factors (such as VEGF) did not clearly point to pro- or antiangiogenic conditions in serum or plasma of TAO patients. Serum of TAO patients and controls was evaluated in proliferation, migration (scratch assay) and spheroid sprouting assays using human umbilical vein endothelial cells (HUVECs). Serum of TAO patients exhibited a diminished sprouting capacity of HUVECs compared to both control groups. Proliferation and migration of endothelial cells were impaired after treatment with serum of TAO patients.

**Conclusion:**

Levels of circulating progenitor cells were altered in TAO patients compared to healthy nonsmokers and smokers. Furthermore, serum of TAO patients exhibited an antiangiogenic activity (impaired endothelial cell sprouting, migration and proliferation) on endothelial cells, which may contribute to vascular pathology in this patient population.

## Introduction

Thromboangiitis obliterans (TAO, also known as Buerger's disease) is a non-atherosclerotic segmental inflammatory vascular disease that primarily affects small and medium sized arteries and veins of the extremities. TAO is observed worldwide with the highest prevalence in the Middle and Far East. Although the disease was first described in 1879 the etiology and pathogenesis of TAO still remains unknown. However, tobacco consumption plays a key role in the initiation and persistence of the disease. TAO typically affects young, male smokers, but the incidence in women is increasing due to tobacco consumption [1], [2].

Generally, intermittent claudication is the first clinical symptom that may progress to critical ischemia with rest pain, digital gangrene and ulcers, finally resulting in amputation of the affected extremity. The prognosis of TAO patients is closely related to smoking. Therefore complete smoking cessation is the most important therapy for TAO and necessary to prevent disease progression and to avoid amputation. Beside the local care of ischemic complications therapeutic options are limited to prostaglandins, anticoagulants, anti-inflammatory agents, immunoadsorption and sympathectomy. In most cases surgical revascularization is not feasible due to the distal location and diffuse vascular occlusions in TAO [2]–[4].

The ischemic condition subsequent to occlusion of the vascular lumen promotes angiogenesis and arteriogenesis leading to the development of collaterals in the affected extremities of TAO patients. A growing number of studies focus on therapeutic angiogenesis as a treatment strategy in patients with coronary artery disease, peripheral arterial disease and also in TAO [5]. In a small clinical trial with TAO patients the intramuscular administration of recombinant *vascular endothelial growth factor* (VEGF) resulted in the healing of ischemic ulcers and relief of rest pain [6]. Increasing evidence suggests that an alteration in stem cell function plays a role in the pathogenesis of vascular diseases [7]. While pilot studies found promising results after autologous transplantation of bone marrow mononuclear cells in TAO patients, little is known about levels of circulating progenitor cells (PC) subsets in TAO [8]–[12]. Therefore, the aim of our study was to evaluate angiogenic processes and factors including circulating progenitor cells in TAO.

## Materials and Methods

### Materials

Unless otherwise specified, all reagents were purchased from Sigma Chemical.

Human umbilical vein endothelial cells (HUVECs) were isolated by collagenase type II (Biochrom KG) digestion of human umbilical veins by means of standard techniques and cultured in endothelial cell (EC) medium (MCDB 131, Gibco-BRL Life Technologies), as described previously [13]. All experiments were performed with HUVECs from passages 1 to 4.

### Study population

For the present study 12 TAO patients were recruited diagnosed on the basis of established diagnostic criteria (onset of disease before the age of 50 years, smoking history, affection of distal arteries, as well as exclusion of atherosclerosis and risk factors other than smoking) with critical limb ischemia: defined as chronic ischemic rest pain and/or evidence of ischemic lesions (either ulcers or gangrene) despite medical therapy. As control groups we enrolled age- and gender-matched nonsmokers (NS, n = 12) and smokers (S, n = 12) without a history of cardiovascular disease. All study subjects were of European descent and had no history of malignancy. The study was approved by the Charité University Hospital Ethics Committee and conforms to the principles outlined in the Declaration of Helsinki. All participants gave written informed consent to participate in this study.

### Study design

All subjects underwent a clinical examination. Blood from the antecubital vein was collected at 8.00 a.m. after a 12-hour overnight fast. Serum and plasma samples were separated from cellular elements by centrifugation and stored at −80°C until analysis. For analysis of progenitor cells, blood was collected in EDTA vials and processed immediately. Routine laboratory tests on blood samples were performed by standard methods in the hospital's laboratory.

### Transcutaneous measurements of tcPO_2_ and tcPCO_2_

To measure tissue oxygenation and carbon-dioxide accumulation, transcutaneous partial oxygen (tcPO_2_) and carbon-dioxide pressure (tcPCO_2_) were determined using a TCM4 device equipped with a Clark electrode (Radiometer®, Copenhagen, Denmark). Measurements were taken at the dorsum of both hands and feet after a resting period of at least 10 minutes, with the patient in supine position in an air-conditioned room maintained at 22°C. The calibration period was 10 minutes and the tcPO_2_/tcPCO_2_ signal was continuously recorded for 30 minutes.

### Flow cytometric quantification of circulating PCs

Circulating progenitor cells were phenotyped and counted using flow cytometry following the protocol suggested by Duda et al. 2007 with modifications [14]. Briefly, undiluted blood samples (0.5 ml) were directly stained and analyzed for phenotypic expression of surface proteins using pre-conjugated anti-human monoclonal antibodies: anti-CD45-PacificBlue (Dako), anti-VEGF receptor-2-PE (R&D Systems), anti-CD34-APC (BD Biosciences), and anti-CD-133-PE (Miltenyi Biotec). Samples were stained with antibodies for 15 minutes in the dark. Erythrocytes were lysed with Easylyse solution (Dako), centrifuged at 200 g for 5 minutes and fixed in 1 ml 0.2% paraformaldehyde in PBS. For each sample, 500.000 events were acquired in the peripheral blood mononuclear cells (PBMC) region using a CyAN-ADP flow cytometer (Beckman Coulter). Data were analyzed using Summit 4.2 software. The PBMCs were identified using their characteristic forward and side scatter profile. CD45^dim^CD34^+^, CD45^dim^CD34^+^CD133^+^, and CD45^dim^CD34^+^VEGFR2^+^ progenitor cells were quantified following the gating strategy described by Duda et al. 2007 [14]. Fluorescence-labeled, isotype-matched nonspecific immunoglobulin G antibodies served as controls for nonspecific staining. All instrument settings and the measurement procedure were stored in a protocol file and remained unchanged throughout the analyses. The gate on the mononuclear cell populations was set individually for each sample. The concentrations of progenitor cells were calculated as “progenitor cells % PBMCs”.

### Levels of circulating angiogenic factors

In the peripheral blood, serum levels of angiopoietin-1, endoglin, endostatin, matrix metalloproteinase-8 (MMP-8) and plasma levels of VEGF were measured using an enzyme-linked immunosorbent assay (R&D Systems). Detection was performed according to the manufacturer's instructions. Each analysis included standard dilutions, and all samples and dilutions were run in duplicate.

### HUVEC proliferation assay

HUVECs were seeded at 8×10^4^ cells/well in 6-well plates and cultured in EC medium. After 24 hours cells from two wells were counted in a hemocytometer and defined as n_1_ at t_1_ = 0 hours. Medium was replaced by fresh EC medium before treatment. EC medium was supplemented with 10% serum of subjects and cells were cultured for additional 24 hours (t_2_). Adherent cells were counted in a hemocytometer (n_2_). Doubling time (t_D_) was calculated as t_D_ = log2 (t_2_−t_1_)/(log n_2_−log n_1_). Supplementation of EC medium with 10% fetal calf serum (FCS) served as positive control. EC medium without serum supplementation was used for negative control. The experiments were performed in duplicate.

### HUVEC spheroid sprouting assay

Spheroids of ∼750 HUVECs were generated as previously described [15]. The HUVEC spheroids were embedded into collagen gels and incubated at 37°C for 24 hours to polymerize. EC medium supplemented with 20% serum of subjects was added to the gel before polymerization started. EC medium without serum supplementation was used as negative control. The core plain of at least 3 spheroids per subject was digitally photographed (Zeiss AxioCam MRc) and in-gel sprouting was analyzed using Zeiss AxioVision software. HUVEC sprouting was quantified as sprouting area surrounding the spheroid and expressed in µm^2^.

### HUVEC migration assay

HUVECs were grown in 24-well plates to a confluent monolayer. The HUVEC monolayer was scraped in a straight line with a 200 µl pipet tip. Cell debris was removed by washing the cells once with 1 ml of the growth medium and followed by incubation with 1 ml of medium supplemented with 10% serum of subjects. A reference point was marked with a tip marker. Dishes were placed under a phase-contrast microscope (microscopic field: 1.4×10^6^ µm^2^) and the first (baseline) image of the scratch was acquired. Dishes were incubated in a tissue culture incubator at 37°C for 6 hours (insufficient time for a significant number of cells to divide). After the incubation, dishes were placed under a phase-contrast microscope (matching the reference point) and a second image was acquired. Images were further analyzed quantitatively by using Zeiss AxioVision software. The area covered by cells per view field before and after 6 hours of incubation was quantified. The difference between areas of the the first and second image was calculated and expressed as migration area in µm^2^. The assay was performed in triplicate.

### HUVEC viability and cell cycle analysis

HUVEC viability and cell cycle analysis were performed by flow cytometry (CyAN-ADP flow cytometer; Beckman Coulter). HUVECs were seeded at 8×10^4^ cells/well in 6-well plates and cultured in EC medium for 24 hours. 10% serum of subjects was added for 24 hours. Cells were trypsinized and resuspended in 400 µl HEPES buffer containing either 10 µg/ml 7-aminoactinomycin D (7-AAD) or 0.05% Triton X-100 and 50 µg/ml propidium-iodide (PI). Cells positive for 7-AAD were defined as nonviable. DNA content of the nuclei was measured by red PI emission for cell cycle analysis. 10.000 events were analyzed for each sample. The experiments were performed in duplicate.

### Statistical Analysis

Data variability about the mean was expressed as the standard error of the mean (SEM), except where otherwise indicated. Statistical analysis was performed by one-way ANOVA with post hoc multiple comparison tests (SPSS v18.0; P<0.05).

## Results

### Baseline characteristics

The baseline characteristics of TAO patients and control groups are shown in Table 1. The mean disease duration of TAO patients at baseline was 5.2±1.3 years. In their most affected extremity TAO patients had elevated tcPCO_2_ (62.3±2.7 mmHg) and remarkably reduced tcPO_2_ (13.6±3.7 mmHg), indicating severely impaired tissue oxygenation.

**Table 1:** Baseline characteristics of the study groups.

	NS(n = 12)	S(n = 12)	TAO(n = 12)
Gender, male/female	9/3	9/3	9/3
Age, years	44.1±4.8	42.6±4.8	44.4±5.1
BMI, kg/m^2^	23.2±3.0	23.7±3.3	26.9±4.4†
WBC, /nl	5.1±1.0	7.7±3.2*	8.5±2.3†
C-reactive protein, mg/dl	0.1±0.1	0.2±0.1	0.8±1.6
Tobacco consumption, pack-years	-	15.5±9.7	26.6±9.3‡
Current smoking, n	-	12	7
Systolic blood pressure, mm Hg	126.7±16.8	141.2±11.9*	130.4±14.0
Diastolic blood pressure, mm Hg	68.6±6.4	78.2±8.2*	73.3±9.5
Total cholesterol, mg/dl	185.6±36.1	198.2±39.4	190.3±54.1
Triglyceride, mg/dl	98.3±57.2	139.3±83.8	151.3±86.7
LDL-cholesterol, mg/dl	113.6±26.9	123.0±40.7	120.3±47.5
HDL-cholesterol, mg/dl	57.9±11.9	49.6±19.2	41.1±8.3†
Diabetes, n	0	0	0
Statin, n	0	0	3

* P<0.05 vs. NS;

† P<0.05 vs. NS;

‡ P<0.05 vs. S; values are mean ± SD; BMI = body mass index; pack-years = (packs smoked per day)×(years as a smoker), 1 pack is considered as 20 cigarettes; HDL = high-density lipoprotein; LDL = low-density lipoprotein; WBC = white blood cell; NS = nonsmokers, S = smokers, TAO = Thromboangiitis obliterans. All results are presented as mean ± SD.

### Levels of circulating progenitor cells and PBMCs in TAO

**Figure 1:**
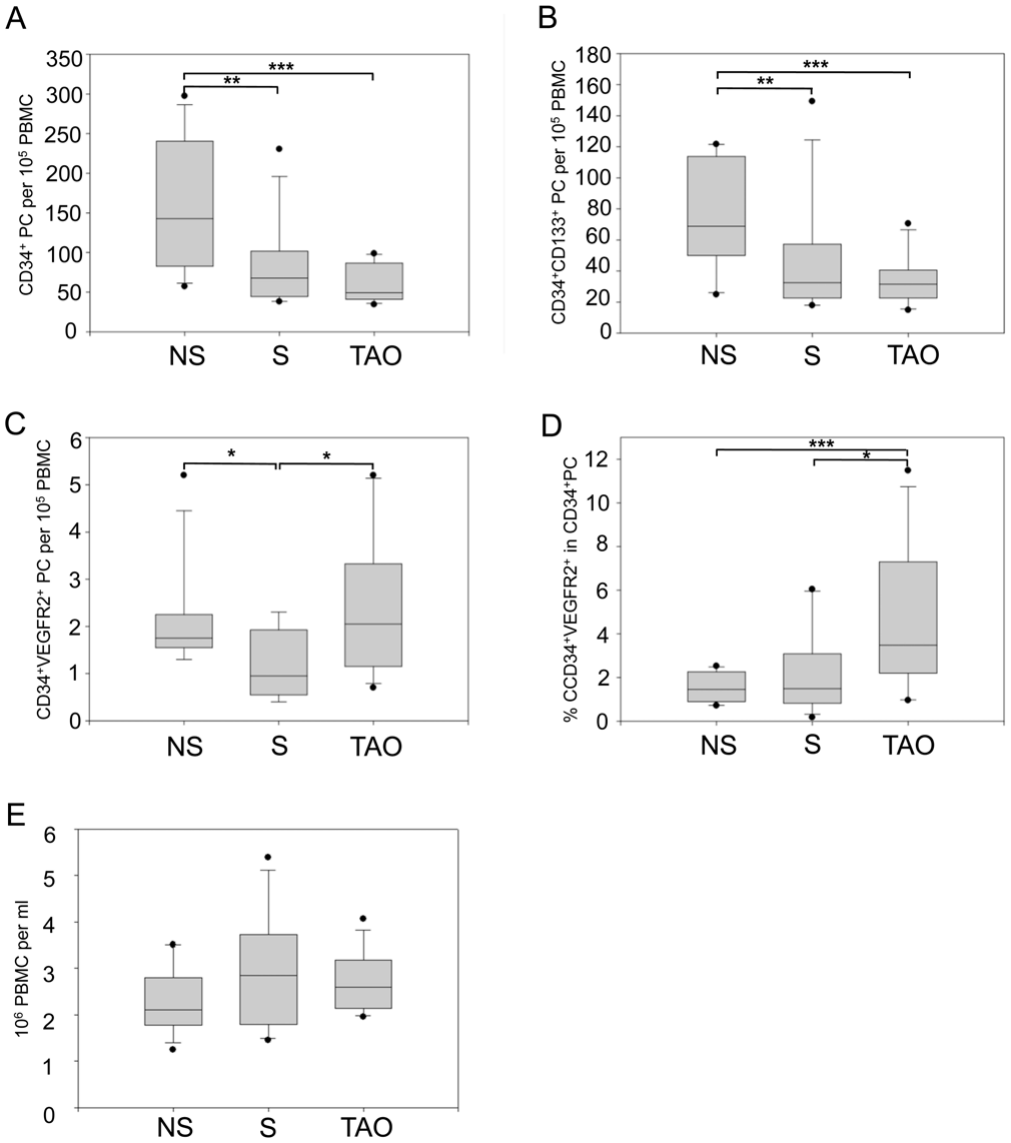
Levels of circulating progenitor cells and PBMCs in TAO patients. Levels of CD45^dim^CD34^+^, CD45^dim^CD34^+^CD133^+^, and CD45^dim^CD34^+^VEGFR2^+^ progenitor cells were measured by flow cytometry. The numbers of progenitor cells (PC) were expressed as cells per 10^5^ peripheral blood mononuclear cells (PBMCs). A) Levels of CD45^dim^CD34^+^ PC, B) levels of CD45^dim^CD34^+^CD133^+^ PC, C) levels CD45^dim^CD34^+^VEGFR2^+^ PC, D) proportion of CD45^dim^CD34^+^VEGFR2^+^ PC on CD45^dim^CD34^+^ PC, E) number of PBMCs per ml of blood. Data are presented as medians, with 25/75 percentiles (boxes) and 10/90 percentiles (bars), (n = 12 in each group). *P<0.05, **P<0.01, ***P<0.001; NS = nonsmokers, S = smokers, TAO = Thromboangiitis obliterans.

We used four surface markers to identify and enumerate the following progenitor cell types by flow cytometry: CD45^dim^CD34^+^, CD45^dim^CD34^+^CD133^+^, and CD45^dim^CD34^+^VEGFR2^+^ progenitor cells. The numbers of progenitor cells were expressed as cells per 10^5^ PBMCs ± SEM. Levels of circulating CD45^dim^CD34^+^ progenitor cells were significantly decreased in smokers as well as in TAO patients compared to nonsmokers (81.2±23.4, P = 0.014 and 60.4±6.8, P = 0.002 vs. 157.3±23.4). TAO patients had slightly lower levels of CD45^dim^CD34^+^ progenitor cells than smokers but the difference did not reach statistical significance (P = 0.236), (Figure 1A). Similarly, CD45^dim^CD34^+^CD133^+^ progenitor cell levels were decreased in smokers and TAO patients compared to nonsmokers (44.4±10.6, P = 0.014 and 34.5±4.7, P = 0.002 vs. 76.2±10.2) with the lowest levels measured in TAO patients (Figure 1B). Also the level of CD45^dim^CD34^+^VEGFR2^+^ cells was significantly reduced in smokers compared to nonsmokers (1.2±0.2 vs. 2.1±0.3, P = 0.028). Strikingly, TAO patients showed a significantly higher level of this subset of progenitor cells compared to smokers (2.4±0.4 vs. 1.2±0.2, P = 0.024), similarly to those of nonsmokers (Figure 1C). This prompted us to calculate the proportion of CD45^dim^CD34^+^VEGFR2^+^ progenitor cells relative to the number of CD45^dim^CD34^+^ cells. Interestingly, in TAO patients the proportion of CD45^dim^CD34^+^VEGFR2^+^ progenitor cells was remarkably elevated compared to both smokers (4.7±1.0% vs. 2.1±0.6%, P = 0.035) and nonsmokers (4.7±1.0% vs. 1.5±0.2%, P = 0.008) (Figure 1D). The average number of PBMCs per ml of blood did not differ significantly between all 3 groups (nonsmokers 2.25±0.21, smokers 2.94±0.36, TAO patients 2.70±0.18×10^6^ PBMCs per ml blood) (Figure 1E).

### Angiogenic factors in serum and plasma of TAO patients

Common angiogenic factors were evaluated in the peripheral blood of TAO patients and both control groups by ELISA (Table 2). There were no significant differences among the 3 groups for serum levels of angiopoietin-1, endoglin, endostatin and MMP-8. Plasma levels of VEGF tended to be higher in TAO patients compared to both control groups, but the differences did not reach statistical significance.

**Table 2:** Angiogenic factors in TAO.

	NS(n = 12)	S(n = 12)	TAO(n = 12)	
Angiopoietin-1 (ng/ml)	45.9±2.9	39.9±2.6	40.9±2.9	n.s.
Endoglin (ng/ml)	5.9±0.2	6.4±0.3	6.1±0.4	n.s.
Endostatin (ng/ml)	93.8±3.6	96.5±2.7	100.3±6.5	n.s.
MMP-8 (ng/ml)	10.5±1.4	16.1±3.2	18.3±4.6	n.s.
VEGF (pg/ml)	49.1±14.1	52.6±8.5	63.2±15.0	n.s.

MMP-8 = matrix metalloproteinase-8, VEGF = vascular endothelial growth factor; NS = nonsmokers, S = smokers, TAO = Thromboangiitis obliterans.

### Serum of TAO patients impairs induction of HUVEC sprouting

**Figure 2:**
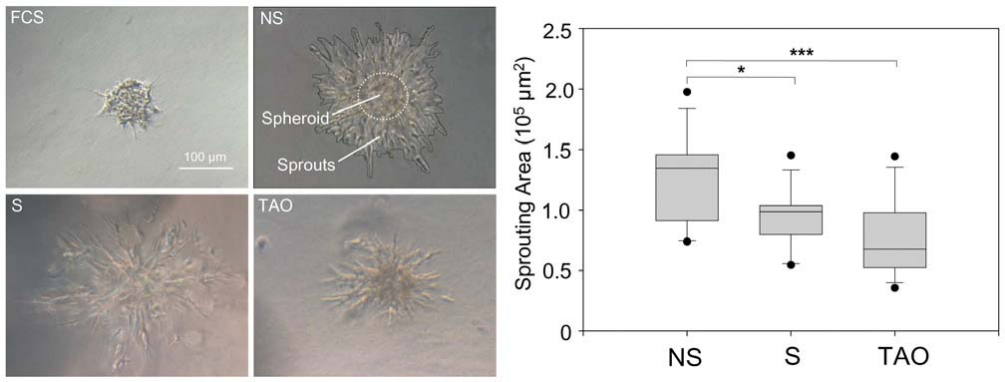
Serum of TAO patients impairs induction of HUVEC sprouting. HUVEC spheroids were embedded in collagen gels supplemented with 20% serum of TAO patients or controls and incubated for 24 hours. Sprouting was quantified as sprouting area surrounding the spheroid (in µm^2^). Data are presented as medians, with 25/75 percentiles (boxes) and 10/90 percentiles (bars), (n = 12 in each group). *P<0.05, ***P<0.001; NS = nonsmokers, S = smokers, TAO = Thromboangiitis obliterans.

An *in vitro* spheroid sprouting assay with HUVECs was used to test the angiogenic effect of serum of TAO patients on endothelial cells. Therefore, HUVEC spheroids were embedded in collagen gels supplemented with 20% serum of TAO patients or controls and incubated for 24 hours. The induction of HUVEC sprouting was impaired in gels supplemented with serum of TAO patients (0.74×10^5^±0.31×10^5^ µm^2^) and smokers (0.94×10^5^±0.24×10^5^ µm^2^) compared to nonsmokers (1.24×10^5^±0.37×10^5^ µm^2^, P = 0.001, P = 0.058, respectively). Serum of TAO patients induced less HUVEC sprouting than serum of smokers, but the difference did not reach statistical significance (P = 0.39) (Figure 2). We also confirmed the antiangiogenic effect of serum of TAO patients using a rat aortic ring assay. Therein, serum of TAO patients induced significantly less vascular cell sprouting compared to serum of nonsmokers and smokers (data not shown).

### Serum of TAO patients inhibits HUVEC migration

**Figure 3:**
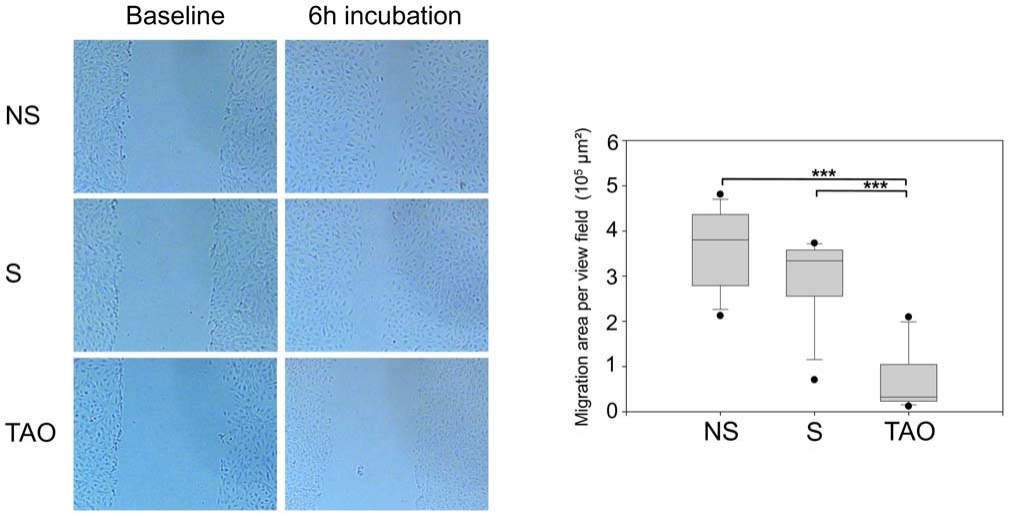
Serum of TAO patients inhibits HUVEC migration. Scratch wound closure assay; a single scrape was made across a confluent HUVEC monolayer (Baseline). The extent of regrowth to close the scratch wound was measured after 6 hours (6 h) incubation in medium containing 10% of serum of TAO patients or controls. Data are presented as medians, with 25/75 percentiles (boxes) and 10/90 percentiles (bars), (n = 12 in each group). ***P<0.001; NS = nonsmokers, S = smokers, TAO = Thromboangiitis obliterans.

A HUVEC scratch assay was used to assess the effect of serum from TAO patients on cell migration. A single scrape was made across a confluent HUVEC monolayer. The extent of regrowth to close the scratch wound was measured after 6 hours incubation in medium containing 10% serum of subjects, an insufficient time for a significant number of cells to divide. The wound closure at 6 hours was significantly reduced with medium containing serum of TAO patients (0.68×10^5^±0.24 µm^2^) compared to nonsmokers (3.62×10^5^±0.24 µm^2^, P<0.001) and smokers (3.02×10^5^±0.24 µm^2^ P<0.001). There was no significant difference in wound closure between nonsmokers and smokers (P>0.05) (Figure 3).

### Serum of TAO patients inhibits HUVEC proliferation by changes in cell cycle progression

**Figure 4:**
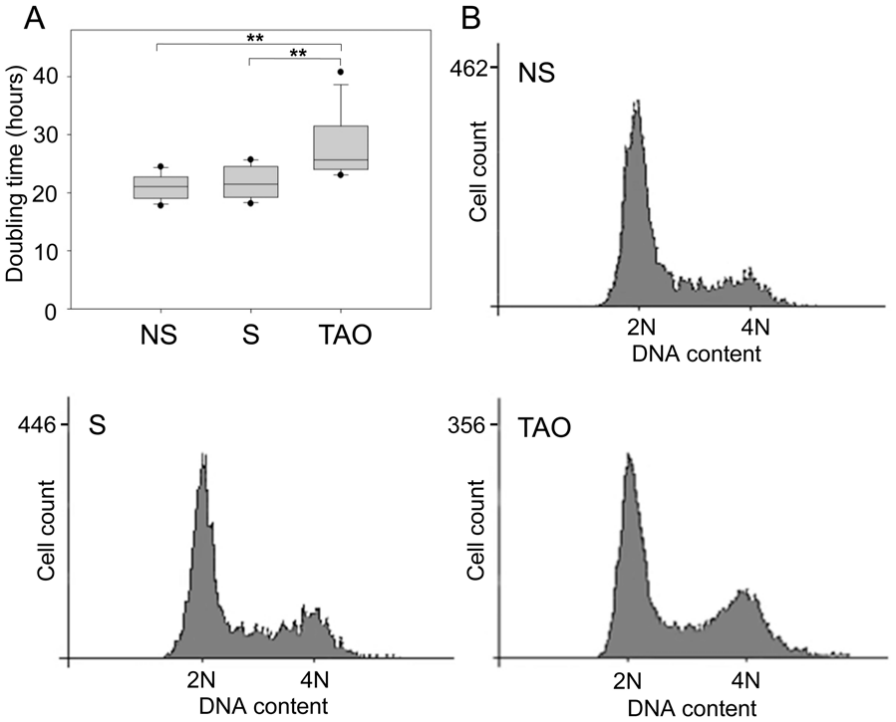
Serum of TAO patients inhibits HUVEC proliferation by changes in cell cycle progression. A) HUVECs were incubated for 24 hours with 10% serum of TAO patients and controls and proliferation was determined as doubling time in hours. Data are presented as medians, with 25/75 percentiles (boxes) and 10/90 percentiles (bars), (n = 12 in each group). *P<0.05, **P<0.01, ***P<0.001. B) For cell cycle analysis HUVECs were cultured for 24 hours with 10% serum of TAO patients and controls, and DNA content was measured by flow cytometry. NS = nonsmokers, S = smokers, TAO = Thromboangiitis obliterans.

Next we tested whether impaired HUVEC sprouting induced by serum of TAO patients is caused by inhibition of endothelial cell proliferation. HUVECs were cultivated in medium supplemented with 10% FCS, before FCS was replaced by 10% serum of TAO patients and controls. HUVEC proliferation was not significantly changed with serum of nonsmokers and smokers compared to FCS, whereas it was impaired with serum of TAO patients. As shown in Table 3 and Figure 4A, the doubling time significantly increased to 27.8±1.5 hours with serum of TAO patients compared to 21.8±0.8 hours with serum of smokers and 21.1±0.6 hours with serum of nonsmokers. Notably, utilizing heat inactivated (56°C for 60 minutes) serum in the proliferation assay increased the doubling time for nonsmokers, smokers and TAO patients to similar levels (31.7±0.6 vs. 32.1±0.7 vs. 30.8±1.4 hours, respectively).

**Table 3:** Proliferation, viability and cell cycle analysis.

	FCserum	NSserum	Sserum	TAOserum	P values
**Doubling time (h)**	22.1±1.1	21.1±0.6	21.8±0.8	27.8±1.5* †	*P = 0.001; †P = 0.001
**Non-viable cells (%)**	3.4±0.3	3.6±0.4	3.3±0.3	3.7±0.4	
**G0/1 phase (%)**	54.3±0.5	54.8±0.9	52.2±0.8	45.4±1.6* †	*P<0.001; †P = 0.001
**S phase (%)**	19.4±0.8	19.8±0.7	20.4±0.6	21.5±0.9	
**G2/M phase (%)**	26.3±0.6	25.3±0.8	27.5±0.4	33.1±0.7* †	*P<0.001; †P<0.001

* TAO vs. NS,

† TAO vs. S; FC = fetal calf, NS = nonsmokers, S = smokers, TAO = Thromboangiitis obliterans.

Next, we performed flow cytometric cell cycle analysis to examine changes in cell cycle progression in HUVECs induced by serum of TAO patients. Cultivation with serum of TAO patients for 24 hours increased the population of HUVECs in the G2/M phase but decreased the population in the G1 phase compared to HUVECs cultivated with serum of nonsmokers and smokers (Table 3 and Figure 4B). No distinct sub G1 peak was observed in any sample, which indicates the absence of apoptosis. Furthermore, 7-AAD labeling revealed that the percentage of non-viable cells was low and nearly the same in HUVECs treated either with serum of TAO patients or with serum of both control groups (Table 3). We also determined the cell size (forward scatter) of HUVECs during the flow cytometric cell viability analysis. The average cell size (arbitrary unit) of HUVECs was significantly increased in TAO patients (153.99±0.58) compared to nonsmokers (150.81±0.41, P<0.01) and smokers (151.40±0.75, P<0.05), reflecting the higher proportion of cells in the G2/M phase. The cell size did not differ significantly between nonsmokers and smokers (P>0.05).

## Discussion

There are two major findings in the present study. First, we found a shift to a higher proportion of CD45^dim^CD34^+^VEGFR2^+^ progenitor cells in TAO patients. Secondly, while angiogenic factors in plasma/serum of TAO patients did not clearly point to pro- or antiangiogenic conditions, serum of TAO patients exhibited a significant antimigratory and antiproliferative effect on mature endothelial cells *in vitro*.

While reduced levels and impaired functional activities of circulating progenitor cells have been described for smokers and patients with cardiovascular disease, there are only a few studies of circulating progenitor cells in TAO patients [7], [11], [12]. In a small study with TAO patients, Katsuki et al. found no significant differences in the levels of CD45^dim^CD34^+^VEGFR2^+^ progenitor cells compared to healthy controls [12]. To our knowledge, there is no study that specifically compares levels of progenitor cells between TAO patients and a control group of smokers. We thought to address this by enrolling two control groups in our study – nonsmokers and smokers without cardiovascular disease – in addition to a TAO patient group, to dissect out the impact of smoking from the disease-related effect on CD45^dim^CD34^+^VEGFR2^+^ progenitor cells.

The overall population of CD45^dim^CD34^+^ progenitor cells, comprising the heterogenic population of hematopoetic stem cell (HSC) and putative endothelial progenitor cells (EPC), were significantly decreased in smokers and TAO patients. While the subset of undifferentiated CD45^dim^CD34^+^CD133^+^ progenitor cells were decreased in smokers and TAO patients, the subset of CD45^dim^CD34^+^VEGFR2^+^ progenitor cells, designated as putative EPCs, were only decreased in smokers but not in TAO patients [16], [17]. Interestingly, although smoking is known to decrease EPCs [18] and severe smoking is common to smokers and TAO patients, levels of CD45^dim^CD34^+^VEGFR2^+^ progenitor cells in TAO patients were similar to levels in nonsmokers. These data imply a disease-related shift to a higher proportion of CD45^dim^CD34^+^VEGFR2^+^ progenitor cells within the CD34^+^ progenitor cell population in TAO patients. It has been shown that the cytokine environment determines the differential mobilization of distinct progenitor cell subsets from the bone marrow. VEGF suppresses the mobilization of hematopoetic stem cells and enhances the mobilization of endothelial progenitor cells via VEGF receptor-2 [19]. The expression of the angiogenic factor VEGF is induced under ischemic conditions [20]. In fact, small clinical studies have shown elevated levels of VEGF in TAO patients consistent with our findings [21], [22]. Accordingly, our data might point to a VEGF-mediated increase in CD45^dim^CD34^+^VEGFR2^+^ progenitor cells mobilization in response to the ischemic disease. Notably, it has been shown that patients with acute myocardial infarction have increased numbers of CD34^+^/VEGFR2^+^ progenitor cells, that correlate with plasma VEGF levels [23]. Pioneering studies about EPCs demonstrated that these cells contribute to tissue regeneration by promoting angiogenesis [16], [24]. So far, a potential regenerative function of endogenous EPCs in patients with TAO has not been validated. Noteworthy, the administration of purified CD34^+^/KDR^+^ progenitor cells promoted vascular regeneration in ischemic limbs of mice and might therefore provide an interesting tool for therapeutic angiogenesis in ischemic diseases [25]. Pilot studies with TAO patients showed promising results after autologous bone marrow mononuclear cell transplantation; however, the contribution of different progenitor cell subsets remains unclear [8]–[10]. Further studies are needed to evaluate the functional consequences of the shift to CD45^dim^CD34^+^VEGFR2^+^ progenitor cells in TAO patients.

Tissue resident endothelial cells play a key role in angio-/arteriogenesis, which is stimulated, at least in part, by circulating factors [26]. To evaluate the angiogenic potential of serum from TAO patients and control subjects, we performed *in vitro* angiogenesis assays using mature endothelial cells. Our data showed that serum from TAO patients exhibits a lower angiogenic capacity as indicated by impaired endothelial cell sprouting compared to serum of controls. Endothelial cell migration and proliferation were decreased after treatment with serum from TAO patients compared to the controls, and were associated with a modulation of cell cycle progression, while cell viability was unaffected. This points to an antimitogenic effect of serum of TAO patients. However, the evaluation of common angiogenic mediators in serum and plasma did not reveal obvious pro- or antiangiogenic conditions in TAO patients. VEGF levels tended to be higher in TAO patients compared to both control groups; but angiopoietin-1, which acts synergistically with VEGF in angiogenesis, did not differ significantly between TAO patients and controls in accordance with a previous publication [12], [27]. Levels of endoglin (CD105), a proangiogenic factor and indicator of human endothelial cell proliferation [28] and endostatin, a potent inhibitor of VEGF effects [29], did not differ significantly between all 3 groups. Taken together, the factor(s) responsible for the decrease in angiogenic potential of serum from TAO patients remain(s) to be identified. However, the fact that heat inactivation of the serums had a more pronounced effect on cell proliferation in the control groups compared to that of TAO patients points to a deficiency or lack of proliferation promoting factor(s) in the serum of TAO patients.

There are some limitations in the present study. The functional and prognostic relevance of the observed shift to higher levels of CD45^dim^CD34^+^VEGFR2^+^ cells in TAO patients, and the functionality of these cells, remain unknown and need to be elucidated in future studies. Furthermore, an *in vivo* angiogenesis model should be used to confirm the data from the *in vitro* angiogenesis assays. Future studies should also evaluate factors and pathways involved in the antimitogenic effect of TAO serum.

In conclusion, TAO patients with peripheral ischemia showed changes in circulating progenitor subsets. Serum from these patients exhibited an antimigratory and antiproliferative effect on mature endothelial cells, which may lead to impaired neovascularization and thus contribute to the acceleration of disease severity. The observations of the present pilot study have to be confirmed in larger studies. These studies, together with further elucidation of angiogenic processes in TAO patients, may provide new insights into the pathomechanism of the disease and thereby contribute to the further development of therapeutic angiogenic strategies.
